# ApoE—functionalization of nanoparticles for targeted brain delivery—a feasible method for polyplexes?

**DOI:** 10.1007/s13346-023-01482-w

**Published:** 2023-12-12

**Authors:** Natascha Hartl, Bettina Gabold, Philipp Uhl, Adrian Kromer, Ximian Xiao, Gert Fricker, Walter Mier, Runhui Liu, Olivia M. Merkel

**Affiliations:** 1 Pharmaceutical Technology and Biopharmaceutics, Ludwig-Maximilians-Universität, Butenandtstr. 5-13, 81377 Munich, Germany; 2 Pharmaceutical Technology and Biopharmaceutics, Ruprecht-Karls-University, Im Neuenheimer Feld 329, 69120 Heidelberg, Germany; 3 State Key Laboratory of Bioreactor Engineering, Frontiers Science Center for Materiobiology and Dynamic Chemistry, Shanghai Frontiers Science Center of Optogenetic Techniques for Cell Metabolism, School of Materials Science and Engineering, East China University of Science and Technology, Shanghai, 200237 China; 4 Department of Nuclear Medicine, University Hospital Heidelberg, Im Neuenheimer Feld 400, 69120 Heidelberg, Germany

**Keywords:** Polyethylenimine, Nylon-3 polymers, siRNA delivery, Polyplexes, Apolipoprotein E, Brain targeting

## Abstract

The blood–brain barrier (BBB) poses a major obstacle in the treatment of all types of central nervous system (CNS) diseases. Small interfering RNA (siRNA) offers in principle a promising therapeutic approach by downregulating disease-related genes via RNA interference. However, the BBB is a formidable barrier for macromolecules such as nucleic acids. In an effort to develop a brain-targeted strategy for siRNA delivery systems formed by electrostatic interactions with cationic polymers (polyplexes (PXs)), we investigated the suitability of the well-known surfactant-based approach for Apolipoprotein E (ApoE)-functionalization of nanoparticles (NPs). The aim of this present work was to investigate if ApoE coating of siRNA PXs formed with cationic branched 25-kDa poly(ethyleneimine) (b-PEI) and nylon-3 polymers without or after precoating with polysorbate 80 (PS 80) would promote successful delivery across the BBB. We utilized highly hydrophobic NM_0.2_/CP_0.8_ nylon-3 polymers to evaluate the effects of hydrophobic cyclopentyl (CP) subunits on ApoE binding efficacy and observed successful ApoE binding with and without PS 80 precoating to the nylon-3 but not the PEI polyplexes. Accordingly, ApoE-coated nylon-3 polyplexes showed significantly increased uptake and gene silencing in U87 glioma cells but no benefit in vivo. In conclusion, further optimization of ApoE-functionalized polyplexes and more sophisticated in vitro models are required to achieve more successful in vitro*-*in vivo translation in future approaches.

**Graphical abstract:**

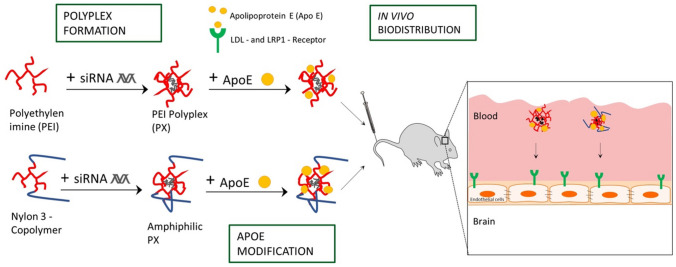

**Supplementary Information:**

The online version contains supplementary material available at 10.1007/s13346-023-01482-w.

## Introduction

The ability of therapeutic agents to reach their target sites in the central nervous system (CNS) is very limited due to the strong protective function of the blood–brain barrier (BBB). However, according to the World Health Organization (WHO), approximately 20% of all humans suffer from CNS disorders such as depression, Parkinson’s disease, Alzheimer’s disease, dementia, epilepsy, stroke, cerebral cancer, or CNS-relevant metabolic diseases. According to recent estimates, the number of people suffering from CNS diseases worldwide will increase significantly in coming decades due to tremendous population growth, increasing life expectancy and in addition, due to risk factors such as obesity, smoking, high blood pressure, or diabetes. Therefore, the development of highly efficient, safe, and targeted pharmaceutical systems that are able to overcome the BBB is urgently needed in order to exert therapeutic effects in disease-specific regions of the brain. Macromolecular drugs, such as proteins, peptides, or nucleic acids, bear a very high potential to open up new perspectives for the treatment of CNS diseases. Less than 2% of small molecule drugs are able to cross the barrier to reach their target sides in the CNS [1]. When it comes to macromolecular drugs, the barrier is completely impermeable due to their high molecular weight [2]. For an extensive review covering transport routes across the BBB, the reader is referred to [3].

A wide range of macromolecular drugs is embedded into nanocarriers to make them stable, safe, and efficient drugs after administration into the human body. In an effort to design efficient nanocarriers that can enable controlled and targeted drug delivery into the brain, a promising option is to decorate the nanocarriers with ligands that can interact with specific binding sites of the BBB with subsequent internalization of the particles via receptor-mediated transcytosis (RMT) into the brain. Among the numerous potential targets at the BBB, the low-density lipoprotein (LDL) receptor family has been extensively investigated as highly efficient target for brain delivery in several studies using liposomes [4] or polymeric nanoparticles (NPs) [3] as drug carriers.

In early attempts, it was found out that some drugs that are commonly unable to cross the BBB can be transported across this barrier and exhibit pharmacological effects after intravenous injection by encapsulating them into poly (butyl cyanoacrylate) (PBCA) NPs. By using PBCA NPs as drug carriers, substances such as the hexapeptide dalargin [5–11], the dipeptide kytorphin [9], loperamide [12], tubocurarine [13], the NMDA receptor antagonist MRZ 2/576 [14], and doxorubicin [15–17] have already been successfully transported into the brain. More continuing studies have confirmed that the brain targeting effect can be further enhanced by precoating the PBCA NPs with surfactants such as PS 20, 40, 60, and 80 [18]. Following the identification of apolipoprotein E (ApoE) on the surface of PS-precoated PBCA NPs after incubation in human plasma [19], Kreuter et al. succeeded in confirming the involvement of plasma ApoE in the brain uptake mechanism of loperamide or dalargin-loaded PBCA NPs (both analgesics) after intravenous injection in mice by measuring the antinociceptive effects with the tail flick test [20].

Since then, numerous small molecule drugs, e.g., methotrexate [21] and cisplatin [22] for the treatment of cerebral cancer or tacrine [23, 24], rivastigmine [25] or rosmarinic acid [26] for treatment of Alzheimer’s disease, and peptide/protein drugs such as the nerve growth factor [27, 28] also for the treatment of Alzheimer’s disease or arylsulfatase A [29] for metachromatic leukodystrophy have been successfully delivered into the brain using PS-coated PBCA NPs. Subsequently, the ApoE targeting approach was successfully applied to several other matrix NPs, e.g., human and bovine serum albumin (HAS [30] and BSA [31]), poly(lactic acid) (PLA) [32, 33], and poly(lactic-co-glycolic acid) (PLGA) [34–37] NPs. Moreover, in addition to the surfactant-based approach, other types of ApoE functionalization of NPs have been investigated such as direct coating of NPs with ApoE, adsorption of ApoE onto NPs by poly(ethylene glycol) (PEG) modification, or covalent linkage of ApoE to NPs.

In an effort to investigate whether the already established brain delivery strategy for solid NPs is also suitable for dynamic systems such as polymer-small interference RNA (siRNA) polyelectrolyte complexes (polyplexes) formed by electrostatic interactions, we evaluated the suitability of the surfactant-based approach with PS 80. The application of siRNA is a promising therapeutic approach in particular for brain tumors due to its ability to downregulate glioma-related genes and to induce tumor growth inhibition, as recently demonstrated in vitro as well as in vivo experiments [38–40]. Since PSs were shown to operate as hydrophobic anchors for binding ApoE, the question arose whether hydrophobic modification of polymers could lead to enhanced ApoE binding without surfactant precoating, what could be beneficial due to concerns and contradictions regarding surfactants’ toxicity [41]. Therefore, besides branched poly(ethyleneimine) (b-PEI) polymers, this study utilized nylon-3 polymers with hydrophobic subunits derived from β-lactam cyclopentyl (CP) and cationic subunits derived from β-lactam without (“no”) methyl substitution (NM) with a subunit ratio of 1:4 (NM:CP), which have been previously investigated regarding efficacy of siRNA delivery into brain tumor cells [42]. Herein, we coated b-PEI and NM_0.2_/CP_0.8_ PXs with ApoE with and without PS 80 precoating and characterized the resulting PXs in terms of their physicochemical characteristics such as particle size, particle size distribution, and surface charge. Sodium dodecylsulfate polyacrylamide gel electrophoresis (SDS-PAGE) was established as a suitable method to verify bound ApoE on PEI and nylon-3 PXs by visualizing them with Comassie Brilliant Blue G staining. Furthermore, cell tolerability, cellular internalization ability, and gene knockdown efficiency of modified and unmodified PEI and nylon-3 PXs were evaluated by CellTiter-Blue^®^ assay, flow cytometry, and real-time quantitative PCR (qPCR) using an LDL and LRP1 receptor expressing model cell line. Since we obtained very promising results in in vitro experiments particularly for ApoE-coated nylon-3 PXs, we first confirmed the hemocompatibility of polymers using a hemolysis assay and subsequently investigated their biodistribution in comparison to b-PEI PXs by measuring the radioactive signals of encapsulated ^177^Lu-labeled DTPA-modified siRNA in the major organs and the brain after intravenous injection in SWISS mice.

## Materials and methods

### Materials

Branched poly(ethylene imine) (b-PEI) 25 kDa, apolipoprotein E (ApoE) from human plasma, HEPES (4-(2-hydroxyethyl)–1-piperazineethanesulfonic acid), Comassie Brilliant Blue G solution, sodium acetate, sodium chloride, sodium hydroxide, sodium hydrogen carbonate, Tween^®^ 80, Dulbecco’s phosphate-buffered saline (PBS), Triton-X, arsenazo(III), yttrium(III) chloride, and for cell culture U87 cells (human glioblastoma astrocytoma), Eagle’s minimum essential medium (EMEM), fetal bovine serum (FBS), penicillin–streptomycin solution, trypsin–EDTA solution 0.25%, and dimethyl sulfoxide (DMSO) were purchased from Sigma-Aldrich (Taufkirchen, Germany). Novex™ 10% tris–glycine gel*,* PageRuler™ Plus Prestained Protein Ladder (10 to 250 kDA), Pierce™ Lane Marker Reducing Sample Buffer, and absolute ethanol were purchased from Thermo Fisher Scientific (Waltham, MA, USA). Gibco^®^ trypan blue solution 0.4% in phosphate-buffered saline was obtained from FisherScientific (Hampton, New Hampshire, USA). Lipofectamin 2000 transfection reagent and AlexaFluor 488 (AF488) dye were purchased from Life Technologies (Carlsbad, California, USA). CellTiter-Blue^®^ Cell Viability Assay was purchased from Promega (Madison, Wisconsin, USA). Rotiphorese^®^10 × SDS-PAGE buffer was obtained from Carl Roth (Karlsruhe, Germany), and mouse ApoE was purchased from Abexxa (Cambridge, UK). 2-(4-Isothiocyanatobenzyl) diethylenetriaminepentaacetic acid (p-SCN-Bn-DTPA) was purchased from Macrocyclics (Dallas, TX, USA) and Hs_GAPDH_1_SG and Hs_ACTB_2_SG QuantiTect primer assays were obtained from Qiagen (Venlo, Netherlands). Fresh human blood was obtained from Blutspendedienst des Bayerischen Roten Kreuzes (Munich, Germany), and EndolucinBeta-Lu^3+^ in aqueous 0.04 M HCl solution was purchased from ITG Isotope Technologies Garching GmbH (Garching, Germany). The following antibodies were used: Anti-LRP antibody, Goat Anti-Mouse IgG H&L (Alexa Fluor^®^ 488) antibody, and Mouse IgG1 kappa monoclonal antibody–isotype control (Abcam, Cambridge, UK). Amine-modified eGFP siRNA (5′-pACCCUGAAGUUCAUCUGCACCACcg, 3′-ACUGGGACUUCAAGUAGACGGGUGGC), human glyceraldehyde 3–phosphate dehydrogenase (GAPDH) siRNA (5′-pGGUCGGAGUCAACGGAUUUGGUCgt, 3′-UUCCAGCCUCAGUUGCCUAAACCAGCA), and scrambled siRNA (5′-pCGUUAAUCGCGUAUAAUACGCGUat, 3′-CAGCAAUUAGCGCAUAUUAUGCGCAUAp) were purchased from Integrated DNA Technologies (Leuven, Belgium). Indication of modified nucleotides: “p” denotes a phosphate residue, lower case letters are 2′-deoxyribonucleotides, capital letters are ribonucleotides, and underlined capital letters are 2′-O-methylribonucleotides.

### Synthesis and characterization of Nylon-3 random copolymer

Nylon-3 copolymer NM_0.2_/CP_0.8_ was synthesized via anionic ring-opening polymerization (ROP) of racemic β-lactams as previously described [42]*.* In brief, monomers β-NM (cationic monomer) [43] and CP (hydrophobic monomer) [44] were prepared according to literature procedures. Random copolymers from β-NM and CP were synthesized following previously reported procedures [45].

### Preparation of polyplexes

To prepare polymer-siRNA complexes (PXs), aqueous b-PEI as well as NM_0.2_/CP_0.8_ polymer stock solutions were diluted with freshly filtered 10 mM HEPES buffer (pH = 7.2) to predetermined concentrations, added to a defined amount of siRNA in a microcentrifuge tube to obtain PXs at various N/P ratios and incubated for 30 min to allow for stable PX formation. The N/P ratio is defined as the molar ratio between the polymer amine groups (N) and the siRNA phosphate groups (P). The amount of polymer needed to obtain different N/P ratios was calculated according to following equation:

$$\begin{aligned}\mathrm m\,\left(\mathrm p\mathrm o\mathrm l\mathrm y\mathrm m\mathrm e\mathrm r\,\mathrm i\mathrm n\,\mathrm p\mathrm g\right)\,=&\,\mathrm n\,\mathrm s\mathrm i\mathrm R\mathrm N\mathrm A\,\left(\mathrm{pmol}\right)\,\times\,\mathrm M\,\mathrm p\mathrm r\mathrm o\mathrm t\mathrm o\mathrm n\mathrm a\mathrm b\mathrm l\mathrm e\,\mathrm u\mathrm n\mathrm i\mathrm t\,\left(\mathrm g/\mathrm{mol}\,\right)\\&\times\,\mathrm N/\mathrm P\times\,\mathrm n\mathrm u\mathrm m\mathrm b\mathrm e\mathrm r\,\mathrm o\mathrm f\,\mathrm n\mathrm u\mathrm c\mathrm l\mathrm e\mathrm o\mathrm t\mathrm i\mathrm d\mathrm e\mathrm s\,\mathrm s\mathrm i\mathrm R\mathrm N\mathrm A\end{aligned}$$

**Scheme 1:**
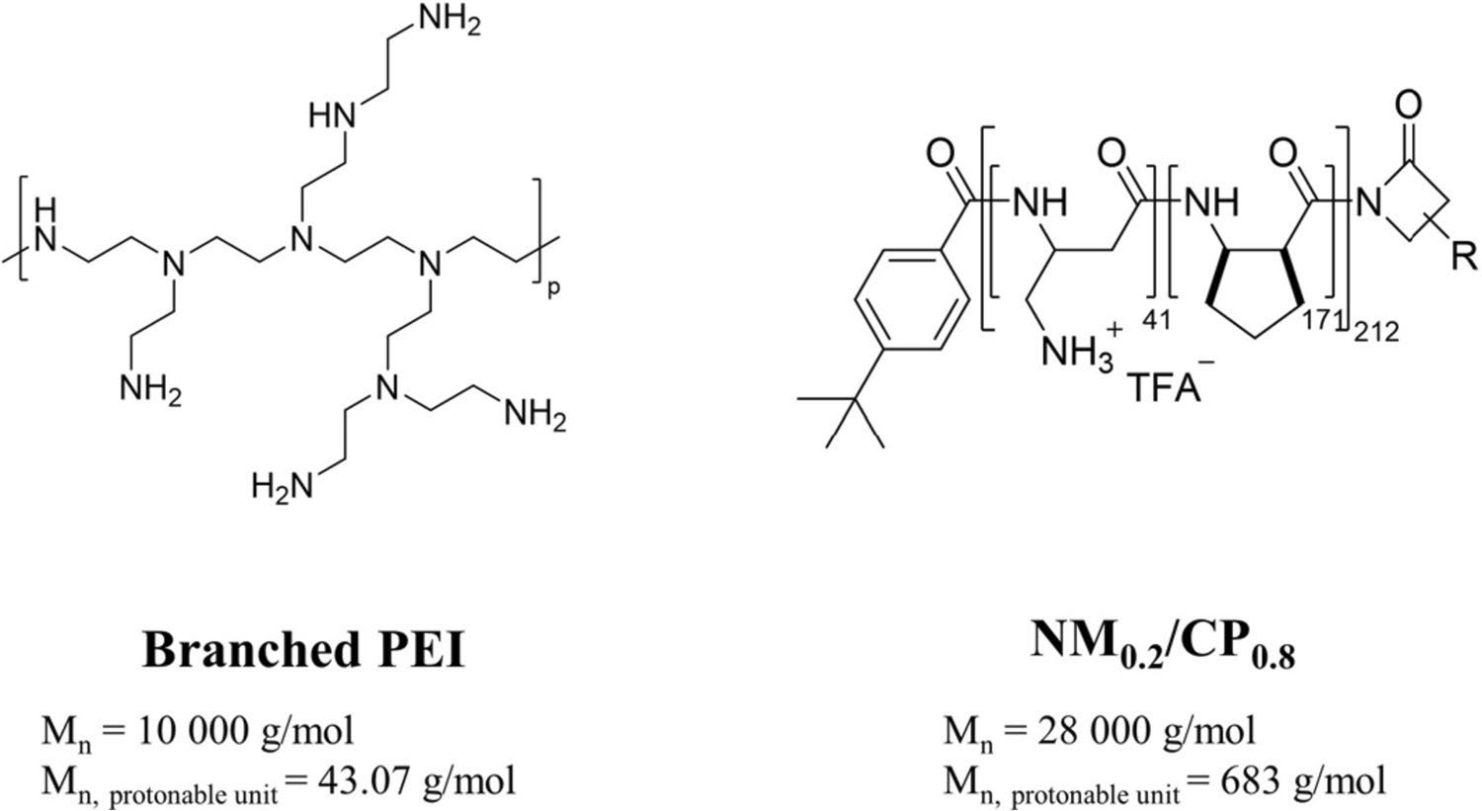
B-PEI and NM_0.2_/CP_0. 8_ polymers used in this study

The protonable unit of each polymer was calculated by dividing its molar mass by the number of protonable primary amines present in each polymer as illustrated in Scheme 1. The number of nucleotides of 25/27mer siRNA is set to 52.

### Coating procedure

For coating of PXs, b-PEI and NM_0.2_/CP_0.8_ PXs were prepared as described above at preassigned N/P ratios. The PXs suspensions were divided into three microcentrifuge tubes; one tube was mixed with PS80 solution 1% to reach a final PS80 concentration of 0.1% (v/v) (PS80 precaoted samples) and the other samples were mixed with the same amount of 10 mM HEPES buffer (pH = 7.2) and stirred at 300 rpm for 30 min. ApoE solution was mixed with the PS80 precoated PX solution (PS80 precaoted and ApoE overcoated samples: PS80 + ApoE-PXs) and with one tube containing plain PXs to reach a final ApoE concentration of 12.5 µg/ml (ApoE coated samples: ApoE-PXs). The third tube was filled up to equal volumes with 10 mM HEPES buffer (pH = 7.2) (uncoated samples used as negative control: NC). Subsequently, all tubes were incubated for 1 h at room temperature (20 – 25 °C) to allow protein adsorption in ApoE samples.

### Size and zeta potential analysis by dynamic light scattering and laser Doppler anemometry

Particle size, polydispersity index (PDI) and zeta potential of PXs were measured using a Zetasizer Nano ZS (Malvern Instruments, Malvern, UK). PXs were formed at an N/P ratio of 8 and subsequently coated as described above. A total volume of 100 µl of each sample was added to a disposal cuvette (Malvern Instruments, Malvern; UK) and used for particle size and PDI measurements by dynamic light scattering (DLS) at 173° backscatter angle running 15 scans three times per sample. Zeta potentials were measured using a Zeta Cell (Zetasizer Nano series, Malvern, UK) containing a 7X dilution of another 100 µl sample aliquot by laser Doppler anemometry (LDA) with each run consisting of 30 scans. Results are expressed as mean ± standard deviation (n = 3).

### Visualization and quantification of bound ApoE by sodium dodecylsulfate polyacrylamide gel electrophoresis (SDS-PAGE)

B-PEI and NM_0.2_/CP_0.8_ PXs were prepared at N/P 8 and coated as described above. PS80 + ApoE-PXs and ApoE-PXs were purified from unbound ApoE analogous to centrifugation procedures optimized for a protein corona—PX approach as previously described in detail [46]. In brief, suspensions were centrifuged at 12.500 g for 10 min, subsequently the supernatant was removed and the pellet was resuspended in 10 mM HEPES buffer (pH = 7.2) followed by an incubation period of 15 min to allow for equilibration. The centrifugation procedure was repeated to ensure removal of unbound ApoE. Samples (uncoated PXs, PS80 + ApoE-PXs, ApoE-PXs) and ApoE reference solution (RS) were mixed with Pierce™ Lane Marker Reducing Sample Buffer and boiled at 95 °C for 5 min to denaturate proteins in a Thermomixer (Eppendorf AG, Hamburg, Germany). A gel electrophoresis chamber Novex^®^ Mini-Cell was prepared with a polyacrylamide-gel 10% placed in tenfold diluted rotiphorese^®^ 10 × SDS PAGE buffer. The slots of the gel were loaded with 5 µl of PageRuler™ Plus Prestained protein ladder (10 to 250 kDA) as molecular marker, 25 µl ApoE RS containing 4.25 µg ApoE as control and 25 µl of NM_0.2_/CP_0.8_ and b-PEI PX samples. The gel was run at 150 mV for 90 min, rinsed twice with deionized water, stained overnight in Comassie Brilliant Blue G solution to visualize the proteins and subsequently destained for 24 h in a mixture of 50% highly purificated water, 40% methanol and 10% acetic acid. A BioRad Chemidoc (Bio-Rad Laboratories, Hercules, CA, USA) was used for image acquisition and densitometric analysis of the gels. Data were processed using Image Lab 6.0.1 software. Results are given as percentage of intensity in comparison to ApoE RS representing 100% intensity.

### In vitro experiments

#### Cells and cell culture

U87 cells (human glioblastoma cell line) were cultured in EMEM media supplemented with heat inactivated FBS (10%) and Penicillin–Streptomycin (1%). All cells were subcultured, maintained and grown in an incubator in humidified air with 5% CO_2_ at 37 °C.

#### Cytotoxicity measurements of PXs by CellTiter-Blue^®^ Assay

Cytotoxicity of PXs was evaluated using a CellTiter-Blue^®^ Cell Viability Assay Kit according to the manufacturer´s protocol based on the ability of living cells to convert a redox dye (resazurin) into a fluorescent end product (resorufin). Nonviable cells rapidly lose metabolic capacity and thus do not generate a fluorescent signal. In brief, 8,000 U87 cells per well were seeded in a transparent 96-well plate (FisherScientific, Hampton, NH, USA) and incubated for 24 h at 37 °C and 5% CO_2_. B-PEI PXs were prepared at N/P ratios of 7 and 15 and NM_0.2_/CP_0.8_ PXs were prepared at N/P ratios of 5 and 15, respectivelyand subsequently coated with ApoE as described above. After consumed medium was completely removed, 90 µL of fresh medium was added to each well and briefly mixed with 10 µL of uncoated PXs or ApoE NM_0.2_/CP_0.8-_PXs solutions. Pure 10 mM HEPES buffer was used as negative control and DMSO 25% in medium was utilized as a positive control. After a 24 h incubation period, 20 µL of CellTiter-Blue^®^ substrate was added to each well followed by another incubation period of 4 h in the incubator. Subsequently, a volume of 100 µL of each sample was transferred to a white 96-well plate (FisherScientific, Hampton, NH, USA) and the fluorescence intensity was measured using a fluorescence plate reader (FLUOstar Omega, BMG Labtech, Ortenberg, Germany) at 560 nm and 590 nm excitation and emission wavelengths, respectively. The experiment was performed in triplicate, and the results are shown as mean ± standard deviation normalized to percentage of viable cells in comparison to untreated cells representing 100% viability.

#### Receptor expression of glioblastoma cells

The expression levels of LRP1 receptors on glioblastoma cells were evaluated by flow cytometry. Therefore, 100,000 U87 glioblastoma cells were transferred into an Eppendorf tube and spun down at 350 g for 7 min and subsequently, the cell pellet was washed with PBS. The cell suspensions were mixed with Anti-LRP1 primary antibody and Mouse IgG1 monoclonal antibody as isotype control to reach a final concentration of 1 µg/ml, respectively. As negative control blank cells without antibody staining were used. All samples were vortexed and incubated for 30 min at 4 °C, while protected from light. Subsequently, cells were spun down at 300 g for 5 min and washed with precooled PBS. After the third washing step, the supernatant was discarded and the cell pellet was resuspended in 100 µl of AF488-labeled secondary goat anti-mouse IgG H&L antibody solution, whereas cell pellets of control samples were resuspended in PBS buffer. After another incubation period of 30 min at 4 °C, cells were washed three times via centrifugation at 400 g for 5 min, resuspended in 400 μl PBS with 2 mM EDTA and analyzed using an Attune R NxT flow cytometer (Thermo Fisher Scientific) by exciting the AF488-labeled secondary antibody at 488 nm and measuring the fluorescence signal with a 530/30 nm emission filter. The cells of all samples were gated according to morphology based on forward/sideward scattering. Samples were run in triplicates, each sample consisting of a minimum of 10,000 viable cells. Results are given as mean ± standard deviation (n = 3).

#### Quantification of cellular uptake into glioblastoma cells by flow cytometry

Flow cytometry was used to quantify the in vitro cellular uptake of b-PEI and NM_0.2_/CP_0.8_ PXs in glioblastoma cells as a function of PS80 + ApoE or ApoE coating. Amine-modified siRNA was labeled with the fluorescent dye Alexa Fluor 488 (AF488) according to the manufacturer´s protocol and purified by ethanol precipitation and spin column binding as described previously [47]. U87 cells were seeded in 24 well plates, and for cell transfection, cell culture medium was replaced with FBS-free culture medium to prevent protein corona formation and thus potentially altered cellular internalization of PXs. B-PEI and NM_0.2_/CP_0.8_ PXs were prepared with 50 pmol siRNA-AF488 at previously optimized N/P ratios for cell transfection, specifically N/P 7 for b-PEI and N/P 5 for NM_0.2_/CP_0.8_ polyplexes and coated with PS80/ApoE as described above. Untreated cells and cells treated with free siRNA were used as negative controls. After a 24 h incubation period, incubation medium was removed, cells were washed with PBS and detached with 0.25% trypsin–EDTA. Samples were washed twice with PBS and resuspended in 500 µl PBS/2 mM EDTA. Additionally, trypan blue quenching was performed to exclude surface fluorescence signals of not completely internalized siRNA-complexes. Median fluorescence intensities (MFI) after quenching were analyzed using an Attune NxT Acoustic Focusing Cytometer (Thermo Fisher Scientific, Waltham, Massachusetts, USA) by exciting the siRNA-AF488 at 488 nm and measuring the fluorescence signal with a 530/30 nm emission filter. Samples were run in triplicates, each sample consisting of a minimum of 10,000 viable cells. Results are given as mean ± standard deviation (n = 3).

#### GAPDH knockdown measurements by real-time quantitative PCR (qPCR)

To investigate gene silencing of b-PEI and NM_0.2_/CP_0.8_ PXs with and without ApoE coating, 300,000 U87 glioblastoma cells per sample were seeded in a 6 well plate (Thermo Fisher Scientific) and grown for 24 h at 37 °C and 5% CO_2._ After changing the medium with fresh FBS-free medium, cells were transfected with b-PEI PXs (N/P 7) and NM_0.2_/CP_0.8_ PXs (N/P 5), containing 100 pmol siRNA either directed against GAPDH (siGAPDH) or scrambled negative control siRNA (siNC), respectively. Samples were coated with ApoE as described above and uncoated samples were used as control. Additionally, Lipofectamine2000 lipoplexes were prepared according to manufacturer´s protocol with siGAPDH and siNC, respectively, and used as positive control. After 48 h incubation, cells were harvested, and total RNA was isolated with the PURELink RNA mini kit (Ambion, Kaufungen, Germany) according to the manufacturer´s protocol with additional DNase digestion. cDNA was synthesized from RNA and amplified with the high-capacity cDNA synthesis kit (Applied Biosystems, Waltham, MA, USA) and QuaniTect primer assays Hs_GAPDH_1_SG and Hs_ACTB_2_SG (Qiagen, Venlo, Netherlands) using a qTOWER real-time PCR thermal cycler (Analytik Jena, Jena, Germany). The RT-qPCR template consisted of an initial denaturation step of 10 min at 95 °C and subsequent 40 cycles at 95 °C for 15 s for further denaturation followed by annealing and elongation step at 60 °C for 1 min. Cycle threshold (Ct) values were obtained with the qPCRsoft software (Analytik Jena), and GAPDH gene expression was normalized by corresponding β-Actin expression for each sample. The relative quantity of transcripts was calculated according to the delta-delta Ct method. Results are given as mean ± standard deviation (n = 3).

#### Hemocompability of polymers measured by hemolysis and erythrocyte aggregation assays

The hemocompatibility and endosomolytic activity of polymers was investigated by red blood cell (RBC) hemolysis and aggregation assays analogous to previous described protocols [48]. Briefly, human erythrocytes were isolated from fresh human blood by centrifugation at 900 g for 10 min. RBCs were washed three times with 150 mM NaCl until the supernatant was clear and colourless. Erythrocytes were again centrifuged at 900 g for 10 min, and the supernatant was replaced with PBS buffer at defined pHs that mimic extracellular (pH 7.4) and late endosomal (pH 5.4) environments. A volume of 10 µl of 20X b-PEI and NM_0.2_/CP_0.8_ polymer solutions in different concentrations (1 mg/ml – 0.0078 mg/ml) was distributed in a 96-well plate. As controls, 20% Triton X-100 (100% lysis) and pure PBS (0% lysis) were used. A volume of 190 µl of the RBC suspension was added to each well, and the plate was incubated for 30 min at 37 °C. RBCs were removed by centrifugation (500 g, 5 min) and supernatant was investigated spectroscopically in a transparent FluoroNunc 96-well plate (FisherScientific, Hampton, NH, USA) by measuring the absorbance of released hemoglobin at 541 nm by using a multimode microplate reader (Tecan Spark, Tecan Group, Männedorf, Switzerland). Measurements were performed in triplicate, and the results are shown as mean values (n = 3). The degree of hemolysis induced by polymers (% hemolysis) was calculated according to the following equation:

$$\mathrm{\%\,Hemolysis}=\frac{\mathrm{Hb}-\mathrm{Hb}_{0}}{\mathrm{Hb_{tot}}}\,\times\,100$$

Hb is the amount of hemoglobin found in the sample, Hb_0_ is the amount of basal hemoglobin found in the negative control samples and Hb_tot_ is the amount of hemoglobin after 100% hemolysis. In addition, to evaluate the aggregation of RBCs after treatment, images of them were taken after the centrifugation step using a Keyence BZ8100 Fluorescence microscope (Keyence, Osaka, Japan) equipped with a Nikon SPLan Fluor 10x/0.45 objective (Nikon, Japan) in the brightfield mode.

### In vivo biodistribution experiments

#### Covalent modification of siRNA with pBn-SCN-Bn-DTPA

In an effort to examine the in vivo biodistribution of b-PEI and NM_0.2_/CP_0.8_ PXs in SWISS mice, siRNA was radioactively labeled with ^177^Lutetium (^177^Lu) following an adjusted protocol previously described by Jones et al. [49]. At first, amine-modified EGFP siRNA (siEGFP) was covalently coupled with the amine-reactive chelator p-Bn-SCN-Bn-DTPA according to a previously described method [50]. Briefly, 5.11 mg siEGFP was dissolved in a centrifuge tube in 2 ml RNase free water, 100 µl of 0.1 M NaHCO_3_ solution were added and siEGFP solution was subsequently mixed with p-Bn-SCN-Bn-DTPA dissolved in DMSO. After thoroughly vortexing the tube and an incubation period of 6 h, siRNA-DTPA was precipitated by adding absolute ethanol. The siRNA-DTPA complex was isolated from free p-Bn-SCN-Bn-DTPA with the absolutely RNA miRNA Kit (Agilent, Santa Clara, CA, USA) according to manufacturer´s protocol. Concentration measurement of siRNA was performed by measuring the absorption at 280 nm with a spectrophotometer (Nanodrop One, Thermo Fisher Scientific, Waltham, MA, USA). The coupling degree after purification was determined by additional performed quantification of DTPA in a nonradioactive assay described by Pippin et al. [51]. Briefly, absorption of an yttrium(III)-arsenazo(III)-complex was measured at 652 nm with a UV/Vis spectrophotometer (UV-1600PC, VWR, Ismaning, Germany), and sample DTPA content was calculated with the help of a calibration curve since the absorption of the complex decreases after addition of DTPA.

#### Labeling and purification

Radiolabeling of the p-SCN-Bn-DTPA-coupled siRNA with ^177^Lutetium (^177^Lu) was accomplished at room temperature in 0.4 M sodium acetate buffer (pH = 5) for 30 min. The siRNA-Lutetium mixture was added to an equilibrated Illustra™ NAP™ 10 column Sephadex G-25 (GE Healthcare, Chicago, ILL, USA) for purification and elution. Fractions were collected and counts per minute were determined by a scintillation counter. The fraction with the highest radioactive signal was additionally investigated by HPLC analysis (Agilent 1100 Series, SEC column: TSK gel Super SW mAb HR) to ensure the presence of siRNA and the absence of free DTPA. The quantification of the siRNA in the final mixture was investigated spectrophotometrically with a UV visible sprectrophotometer (Cary 50 Conc, Varian) by measuring the absorbance at 260 nm.

#### In vivo biodistribution

All animal trials were approved by the Animal Care and Use committees at the University of Heidelberg, Heidelberg, Germany and the responsible government agency (Regierungspräsidium Karlsruhe, Germany, reference number 35–9185.81/G-111/16; approval date: 22 June 2016). For in vivo experiments, polyplexes with ^177^Lu-labeled siRNA were prepared at N/P ratio of 7 with b-PEI polymer and at N/P ratio of 5 with NM_0.2_/CP_0.8_ polymers, respectively. One part of NM_0.2_/CP_0.8_ PX was additionally coated with mouse ApoE as described above. Free ^177^Lu-labeled siRNA as control and PXs samples were injected intravenously to the tail vein of SWISS mice (2 nmol siRNA/animal), and biodistribution was investigated 1 h post injection. To this end, the animals were sacrificed, major organs were removed and weighed and the radioactivity of each sample was measured using a Cobra Auto γ-Counter (Packard BioScience Co., Meriden, CT, USA) in comparison with standards. The tissue-associated activity was related to the total injected dose (ID) and calculated as a percentage of the total injected dose per gram of the respective organ (ID%/g).

### Statistics

Unless otherwise stated, results are given as mean value ± standard deviation. One-way ANOVA with Bonferroni multiple comparison test and two-way ANOVA were performed in GraphPad Prism software (Graph Pad Software, La Jolla, CA) to calculate p-values at 95% confidence.

## Results and discussion

### Synthesis and characterization of nylon-3 random copolymer

NM_0.2_/CP_0.8_ polymer was prepared with hydrophobic and hydrophilic β-lactams via anionic ring-opening polymerization (ROP) as described above. The synthesis led to a nylon-3 copolymer that contains randomly arranged hydrophobic and cationic subunits in a 1:4 ratio. The hydrophobic monomer was cyclopentadienyl β-lactam (CP), and the cationic monomer was a β-lactam without a methyl group (NM). Molecular weight and subunit ratios were determined by ^1^H-NMR spectroscopy as reported previously [42]. In this study, performed by our group, suitability for siRNA delivery into glioblastoma cells in comparison to various other nylon-3 polymers has been described and NM_0.2_/CP_0.8_ polymer was shown most promising regarding siRNA delivery into glioblastoma cells due to the high hydrophobic content [42]. Furthermore, since it was shown that hydrophobic moieties are able to induce adsorption of ApoE, the highly hydrophobic NM_0.2_/CP_0.8_ polymer was selected in this study to investigate the ApoE adsorption on NM_0.2_/CP_0.8_ PXs in comparison to cationic b-PEI PXs in an effort to examine the influence of ApoE adsorption on siRNA delivery efficiency of PXs across the BBB.

### Size and zeta potential analysis of PXs by dynamic light scattering and laser Doppler anemometry

In order to investigate the hydrodynamic diameters, polydispersity indices, and zeta potentials of uncoated particles in comparison to coated PXs, DLS and LDA measurements were performed. Particle size and charge are two major parameters of NPs that affect intracellular uptake and transfection ability. Therefore, the first step of our study was to investigate the influence of modifying the particles with ApoE or PS80 + ApoE on the physicochemical characteristics of the PXs. For PS80 coating, we initially tested the optimal PS80 concentration by incubating the PXs with various PS80 concentrations ranging from 0.01 to 0.5% PS80 with subsequent measurement of sizes and PDIs of PS80-PXs by DLS. As illustrated in Fig. S1 (Supplementary Material), the sizes of NM_0.2_/CP_0.8_ PXs sizes were not affected at all by PS80 coating at the selected concentrations, whereas b-PEI PXs were destabilized by PS80. Only at PS80 concentrations of 0.1% and higher, particle sizes with low PDI values, which were comparable to those of the reference particles were obtained. In the case of NM_0.2_/CP_0.8_ PXs, we suggest that interactions between hydrophobic CP parts of the polymer and oleic acid groups of PS80 molecules resulted in immediate surface coating, reflected by colloidally stable particles over a wide PS80 concentration rage. However, in the case of b-PEI, it is conceivable that low surfactant concentrations led to destabilization and aggregation due to interactions between cationic amino groups of the PEI molecule with carboxyl groups of the PS80 molecule before the optimal concentration for polyplex stabilization was reached. Moreover, excess surfactant at concentrations above the optimum contributed to self-assembly of the molecule chains in micelles, which increased the PDI values in the DLS measurement [52]. Therefore, 0.1% PS80 was chosen as optimal concentration for further experiments.

**Fig. 1:**
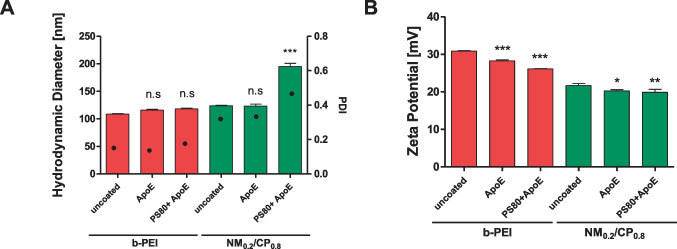
Hydrodynamic diameters (left *y*-axis) and polydispersity indices (PDI, right *y*-axis) (**A**) and zeta potentials (**B**) of uncoated PXs, ApoE PXs, and PS80 + ApoE PXs formed with b-PEI and NM_0.2_/CP_0.8_ polymers at N/P ratio of 7 and 5, respectively (data points indicate mean ± SD, *n* = 3, one-way ANOVA with Bonferroni post hoc test, n.s not significant, *p < 0.05, **p < 0.01, and ***p < 0.005)

For b-PEI PXs, as shown in Fig. 1, coating with ApoE and PS80 + ApoE resulted in a slight increase in particle size from 108.6 nm for the uncoated sample to 116.0 nm and 118.2 nm for the ApoE-coated sample and the PS80 + ApoE-coated sample, respectively. The PDI values were in the same range for all b-PEI samples; in detail, they amounted 0.095 for uncoated b-PEI PXs, 0.081 for ApoE b-PEI PXs, and 0.120 for PS80 + ApoE b-PEI PXs. The finding for the uncoated b-PEI PXs is in line with previously published results [53, 54]. The uncoated and ApoE-coated NM_0.2_/CP_0.8_ PXs revealed similar sizes of 123.9 nm and 123.3 nm, respectively, whereas the size for PS80 + ApoE-coated PXs increased in comparison to other samples to 194.8 nm. The same trend was also observed for the PDI values of NM_0.2_/CP_0.8_ PXs as they increased from 0.260 (uncoated PXs) and 0.272 (ApoE-coated PXs) to 0.401 for PS80 + ApoE-coated samples. The finding for uncoated NM_0.2_/CP_0.8_ PXs is in line with former studies [42]. The zeta potential of uncoated b-PEI PXs was + 30.9 mV and decreased with ApoE coating to + 28.23 mV and to + 26.10 mV after PS80 + ApoE coating. Uncoated NM_0.2_/CP_0.8_ PXs showed a zeta potential of + 21.7 mV that also slightly decreased after ApoE and PS80 + ApoE coating to + 20.27 mV and + 19.9 mV, respectively. All DLS measurements were performed in buffer, which does not reflect natural protein corona formation but allowed for investigating the impact of coating, washing, and centrifuging the PXs.

Since human ApoE has an isoelectric point of 5.55 [55] and is thus negatively charged at a pH of 7.2, it was concluded that ApoE molecules are adsorbed on the surface of positively charged PXs by electrostatic interactions and, in the case of NM_0.2_/CP_0.8_ PXs, additionally by hydrophobic interactions what might lead to the measurable size increase. ApoE is responsible for the redistribution of lipids among cells and tissues as parts of lipoproteins in the body and therefore bears a lipid binding region in the C-terminal domain [56]. For NM_0.2_/CP_0.8_ PXs, precoating with PS80 resulted in a higher and statistically significant size increase than for b-PEI PXs (no significant change), which might occur due to more efficient interactions between PS80 and NM_0.2_/CP_0.8_ PXs. The significant zeta potential decrease after coating with ApoE and PS80 + ApoE supports the assumption that negatively charged ApoE is adsorbed on the PXs’ surface. In summary, coating of ApoE and PS80 + ApoE showed little effect on the particle size, size distribution and zeta potentials of the PXs, resulting in particles with appropriate sizes and surface charges for further experiments.

### Evaluation of bound ApoE by sodium dodecylsulfate polyacrylamide gel electrophoresis (SDS PAGE)

SDS-PAGE is a commonly used electrophoretic technique for separation and analysis of proteins based on their molecular weight. Protein bands within the gel can be visualized by a colorimetric staining such as Comassie [57]. SDS-PAGE was hence performed to investigate whether and to which extent ApoE is indeed adsorbed on different formulations. Therefore b-PEI and NM_0.2_/CP_0.8_ PXs were coated with ApoE and PS80 + ApoE, respectively, as described above. Coated PXs were purified via centrifugation as described above to remove free and unbound ApoE. Purified PXs were resuspended after the last centrifugation step and 25 µl of each sample (uncoated, ApoE-coated, and PS80 + ApoE-coated b-PEI and NM_0.2_/CP_0.8_ PXs) and ApoE RS (34.2 kDa) as reference, were loaded and run on a polyacrylamide gel for visualization of proteins by staining with Comassie Brilliant Blue G.

**Fig. 2:**
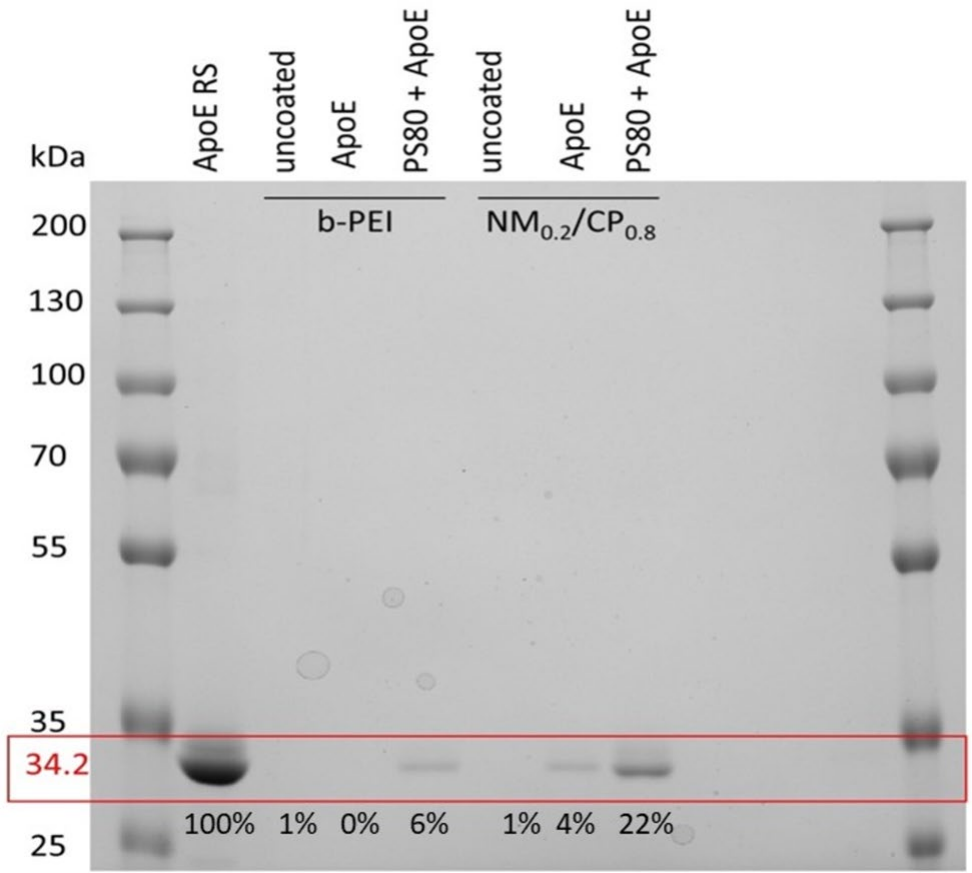
ApoE bound to NM_0.2_/CP_0.8_ and b-PEI polyplexes with and without PS80 precoating evaluated by SDS-PAGE performed with a polyacrylamide-gel 10%. The slots of the gel were loaded with a molecular marker (10 to 250 kDa), ApoE RS (34.2 kDa) as reference, uncoated PXs, ApoE-PXs, and PS80 + ApoE-PXs samples. Proteins were visualized by staining with Comassie Brilliant Blue G. Gel was analyzed densitometrically and results for ApoE content are given in percentage of intensity in comparison to ApoE RS representing 100% intensity

As displayed in Fig. 2, no bound ApoE was detected by SDS-PAGE on ApoE-coated b-PEI PXs (0%), whereas a slight band of ApoE was visible for PS80-precoated b-PEI PXs (6%). For NM_0.2_/CP_0.8_ PXs a slight band of ApoE appeared for ApoE-coated NPs (4%), while a more distinct ApoE band was observed for PS80-precoated samples (22%), indicating that a larger amount of ApoE was attached to PS80 + ApoE NM_0.2_/CP_0.8_ PX samples. These results are supported by the more visible size increase of PS80-precoated samples and led to the conclusions that the coating of PXs with ApoE was generally possible but the binding affinities of ApoE strongly depend on the underlying NP material and its modification, which is in line with previous reports. A former study of Blank assessed bound proteins on model polystyrene carriers as a function of their modification after incubation in plasma and described that on hydrophobically modified particles mainly apolipoproteins were found [58]. Subsequently, it was described for a wide variety of NPs that hydrophobicity facilitates the binding of specific proteins, such as ApoE [59]. Herein, we suggest that the C-terminal domain of the ApoE molecule that contains a lipid binding region [60] can directly interact with the hydrophobic CP subunits of the NM_0.2_/CP_0.8_ PX. Moreover, several other studies described that successful binding of ApoE was achieved by a surfactant-based approach in which poloxamers or PS were used as hydrophobic anchor [3]. Our results for PXs are in line with the literature, as adsorption of ApoE was not detected on cationic b-PEI PXs, while ApoE was found, albeit to a small extent, on more hydrophobic NM_0.2_/CP_0.8_ PXs. Moreover, PS80 precoating led for both, b-PEI and NM_0.2_/CP_0.8_ PXs to an adsorption of ApoE. Herein, we hypothesize that the higher amount of ApoE on NM_0.2_/CP_0.8_ PXs can be explained by more efficient interactions with PS80. PS80 might adsorb with its hydrophobic part (oleic acid) to the CP subunits of the NM_0.2_/CP_0.8_ PXs, whereas the hydrophilic part ((poly(ethylene oxide) (PEO) sorbitan) protrudes into the dispersion medium and facilitates the binding of ApoE, as similarly described for poloxamers by the group of Blunk et al. [58]. Taken together, SDS-PAGE results illustrated that functionalization of PXs with ApoE was successful by direct coating of NM_0.2_/CP_0.8_ PXs and by utilizing the surfactant-based approach with PS80 for both, b-PEI and NM_0.2_/CP_0.8_ PXs.

### In vitro experiments

#### Quantification of cellular uptake into glioblastoma cells by flow cytometry

**Fig. 3:**
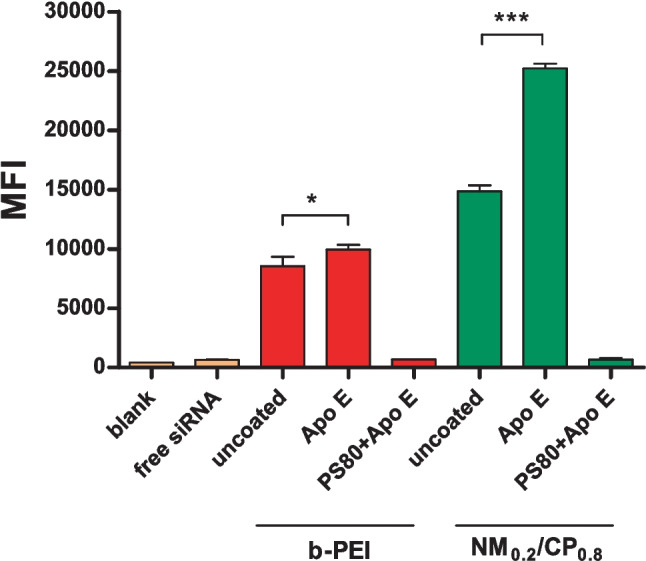
Cellular uptake of uncoated PXs, ApoE PXs, and PS80 + ApoE PXs (b-PEI PXs: N/P ratio = 7 and NM_0.2_/CP_0.8_ PXs: N/P ratio = 5) after 24-h incubation as quantified by flow cytometry performed with trypan quenching and presented as median fluorescence intensitiy (MFI). Negative control: untreated cells (blank) and with free siRNA-treated cells (results are shown as mean ± SD, *n* = 3, one-way ANOVA with Bonferroni post hoc test; *p < 0.05 and ***p < 0.005)

As successful coating of PXs with ApoE was confirmed, the next step was to investigate whether this may lead to selective uptake of NPs in an LDL and LRP1-receptor bearing human glioblastoma cell line. ApoE is a component in lipoprotein classes VLDL and chylomicrons and mediates lipid transport and delivery into cells mainly via LDL and LRP1 receptor-mediated pathways [61]. The N-terminal domain of the ApoE molecule contains a lysine- and arginine-rich receptor binding site, and interactions of this domain with the respective receptor initiate endocytotic uptake of the particles into cells [62]. LDL receptor expression of the U87 glioblastoma cell line used in this study was measured elsewhere [63] and expression of LRP1 receptors was confirmed by antibody staining and subsequent flow cytometry measurements as described above and shown in Fig. S2 (Supplementary Material). Trypan blue quenching, which was additionally performed in order to exclude extracellular fluorescent signals caused by cell surface-bound siRNA, resulted in insignificantly lower MFI values for all tested b-PEI PXs, indicating that inconsiderable amounts of PXs were only bound to the outer cell membranes, whereas significantly lower MFIs values were detected for all tested NM_0.2_/CP_0.8_ PXs, pointing out that NM_0.2_/CP_0.8_ PXs s adhered more strongly to the cells due to hydrophobic interactions (Fig. S3, Supplementary Material). In order to exclude signals caused by surface-bound siRNA when quantifying cellular uptake of formulations, MFI values are presented after trypan quenching. As illustrated in Fig. 3, PS80-coated particles showed, against our expectations, no differences in MFI values in comparison to negative control samples with free siRNA and therefore no measurable cellular internalization into glioblastoma cells. The MFI value for negative control was 646.5, for PS80 + ApoE- b-PEI PXs 680.1 and for PS80 + ApoE-NM_0.2_/CP_0.8_ PXs 651.8, respectively. Uncoated NM_0.2_/CP_0.8_ PXs (MFI = 14,852) revealed a slightly higher cell entry capability than b-PEI PXs (MFI = 8548), what goes in line with literature [64]. The most efficient siRNA delivery in glioblastoma cells was observed by the ApoE-coated PXs, where significantly increased cellular uptake was detected in comparison to the uncoated particles. This effect, however, was even more pronounced for NM_0.2_/CP_0.8_ PXs than for b-PEI PXs. The MFI value for ApoE-coated b-PEI PXs was 9957 (uncoated 8548) and for ApoE-coated NM_0.2_/CP_0.8_ PXs an average MFI value of 25,216 (uncoated 14,852) was observed.

The obtained results regarding the cellular uptake of PS80 + ApoE PXs are not in line with our expectations that arose from the SDS-PAGE result, which indicated successful binding of ApoE on PXs especially on PS80-precoated particles as presented in “Evaluation of bound ApoE by sodium dodecylsulfate polyacrylamide gel electrophoresis (SDS PAGE).” As no cellular uptake was detected for both, b-PEI and NM_0.2_/CP_0.8_ PS80-precoated samples, it may be speculated that PS80-ApoE-modified PXs were destabilized in the complex environment of cell culture medium that was required for the uptake experiment, so that no functional particles were present to enter the cells. To exclude the possible influence of ApoE coating, we repeated the experiment exemplarily using PS80-precoated b-PEI PXs and uncoated b-PEI PXs. As displayed in Figure S4 (Supplementary Material), PS80-precoated b-PEI PXs were not able to enter glioblastoma cells in comparison to uncoated b-PEI PXs confirming that PS80 coating tremendously reduced the cellular uptake ability of PXs. Our hypothesis that in particular hydrophobic NM_0.2_/CP_0.8_ PXs constitute a promising system for the PS80-ApoE targeting approach that were supported by promising SDS-PAGE and DLS results was hereby disproved. Consequently, PS80 coating does not seem to be a suitable approach for PXs in general, whereby the exact impact of PS80 coating on PX destabilization remains unclear and gives rise to investigate this question in future studies. Consequently, PS80-precoated samples were not considered further in the following experiments. Notwithstanding, the uptake data revealed that modification of PXs with ApoE alone significantly enhanced the cellular internalization ability of both b-PEI and NM_0.2_/CP_0.8_ PXs, whereas this effect was more pronounced for NM_0.2_/CP_0.8_ PXs. Therefore, we suggest that adsorbed ApoE on the surface of the PXs interacted with LDL and LRP1-receptors and consequently induced selective receptor-mediated endocytosis of PXs into glioblastoma cells [65]. This goes in line with recently described data in the literature. Particularly, the group of Kreuter et al. has described that coating of analgesic hexapeptide dalargin-loaded PBCA NPs with ApoE or ApoB alone mediated the delivery of the cargo across the BBB after intravenous injection. However, in their studies, ApoE/B overcoating after PS80 precoating induced an even higher effect in the in vivo experiment [20]. Further, the group of Mulik et al. has shown that PBCA NPs loaded with the drug curcumin were more efficiently taken up by SH-SY5Y cells after coating them with ApoE in comparison to plain PBCA NPs [66, 67]. These results underline our finding that ApoE coating of NPs alone could induce receptor-mediated endocytosis and that this approach can be translated from solid NPs to dynamic systems such as PXs at least in an in vitro setting. Interestingly, and in line with SDS-PAGE results, ApoE-NM_0.2_/CP_0.8_ PXs were more efficiently taken up in comparison to ApoE-treated b-PEI PXs what may be an indication that a higher amount of ApoE bound on NM_0.2_/CP_0.8_ PXs is able to induce more efficient receptor-mediated cellular uptake. In conclusion, our results indicate that a surfactant-based targeting approach with PS80 precoating for enhanced ApoE binding does not constitute an appropriate strategy for dynamic systems such as PXs due to suspected destabilization under in vitro conditions. However, direct coating of PXs with ApoE resulted in remarkably increased cell uptake, particulary for NM_0.2_/CP_0.8_ PXs, rendering them into highly promising candidates for ApoE-induced brain delivery. Considering the high LRP-1 expression of U87 cells, the ApoE-dependent uptake is hypothesized to be receptor-mediated.

#### GAPDH knockdown measurements by PCR

**Fig. 4:**
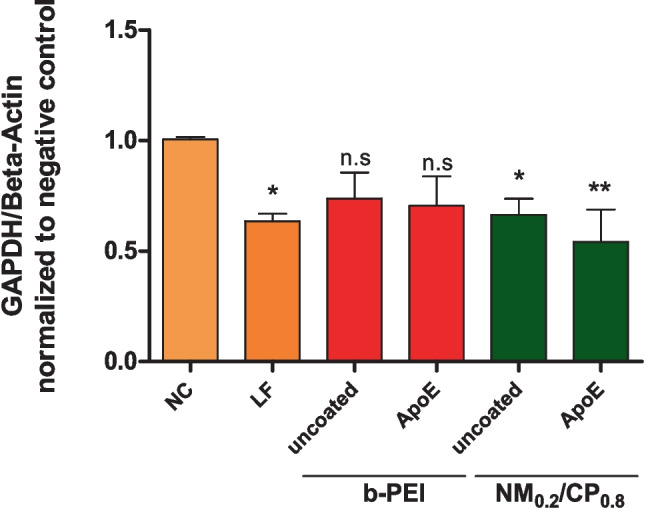
GAPDH knockdown in the human glioblastoma cell line U87 as quantified by RT-PCR 48 h after transfection with uncoated and ApoE-coated b-PEI and NM_0.2_/CP_0.8_ PXs prepared with GAPDH siRNA or scrambled control siRNA as respective negative control (NC). The positive control consisted of Lipofectamine (LF) 2000 lipoplexes. The expression of GAPDH was normalized to the expression of β-actin and to cell samples transfected with the respective negative control PX (data points indicate mean ± SD, *n* = 3, one-way ANOVA with Bonferroni post-hoc test, n.s not significant; *p* > 0.05, *p < 0.05, and **p < 0.01)

Next, we investigated whether the significantly higher internalization of ApoE-modified PXs correlated with the gene silencing of a targeted gene. Therefore, glioblastoma cells were transfected with ApoE-b-PEI PXs and ApoE-NM_0.2_/CP_0.8_ PXs prepared with siRNA against GAPDH (siGAPDH) or scrambled negative control siRNA (siNC), respectively. Uncoated samples were used as negative control, and Lipofectamine 2000 lipoplexes were utilized as positive control samples. GAPDH gene expression was quantified by real time PCR (RT-qPCR) and normalized by β-actin gene expression and normalized to the values obtained after transfection with negative control siRNA for each sample. As displayed in Fig. 4, significant downregulation of GAPDH expression was observed for uncoated NM_0.2_/CP_0.8_ PXs (knockdown of 34%) and more pronouncedly for ApoE-NM_0.2_/CP_0.8_ PXs (knockdown of 46%) compared with negative control samples. In contrast, there was no significant difference of GAPDH expression among b-PEI PX (uncoated and ApoE-coated)-treated cells and their respective negative controls. Positive control samples displayed a GAPDH gene silencing effect of 36% for Lipofectamine lipoplexes, reflecting the poor transfectability of U87 glioblastoma cells in general.

Unmodified b-PEI PXs can undergo endocytosis by electrostatic interactions between their positive surface charge and the negative charge of the cellular membrane, however, the internalization of b-PEI PXs was not sufficient to induce significant GAPDH gene silencing effects. A lack in GAPDH knockdown ability was also observed for ApoE modified b-PEI particles, implying that even receptor-mediated endocytosis of b-PEI particles did not lead to significant gene knockdown effects. This goes in line with literature, as high molecular weight b-PEI was shown to exhibit, besides low toxicity, poor transfection efficiencies [68]. In contrast, unmodified NM_0.2_/CP_0.8_ PXs demonstrated efficient gene knockdown, leading to the assumption that additional hydrophobic interactions with the outer cell membrane as well as with the endosomal membrane might have beneficial effects on the siRNA amount present in the cytoplasm, what is a prerequisite for successful gene silencing. As described in literature, endosome disruption can be caused by cationic as well as hydrophobic moieties of polymeric NPs, which are both present in NM_0.2_/CP_0.8_ polymers [69]. However, in accordance with cellular uptake results, ApoE modification of NM_0.2_/CP_0.8_ PXs mediated the most efficient gene knockdown effect, comparable to the commercially available transfection reagent Lipofectamine 2000, indicating that receptor-mediated endocytosis in combination with favorable characteristics of the NM_0.2_/CP_0.8_ PXs provided most suitable conditions to induce sequence-specific gene silencing in a cell line, which is generally described to be hard to transfect [42]. Overall, the results presented here demonstrated efficient knockdown ability of ApoE-modified NM_0.2_/CP_0.8_ PXs, providing further evidence for the applicability of the targeted siRNA delivery system for potential treatment of brain diseases.

#### Cytotoxicity measurements

##### Cytotoxicity measurements of PXs by CTB assay

**Fig. 5:**
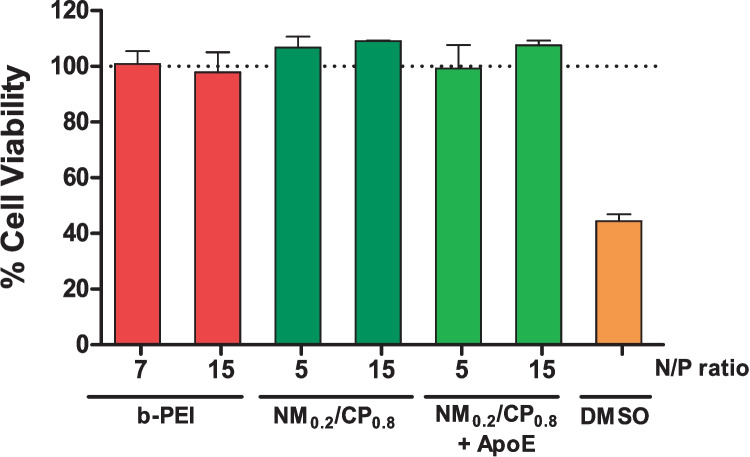
Cell viability as determined by CTB assay for formulated polyplexes at relevant N/P ratios after an incubation period of 24 h. DMSO 25% was used as positive control (Results are shown as mean ± SD as percentage of viable cells in comparison to untreated cells representing 100% viability, *n* = 3)

In order to test the cytotoxicity of PXs, CTB assays were conducted with U87 cells that had been incubated for 24 h with uncoated b-PEI and NM_0.2_/CP_0.8_ PXs and ApoE NM_0.2_/CP_0.8_ PXs at two different N/P ratios. Thereby, lower N/P ratio of 5 (b-PEI) and 7 (NM_0.2_/CP_0.8_ PXs) represented treatment relevant conditions in in vitro experiments. The CTB assay is based on the ability of viable cells to reduce the nonfluorescent resazurin to fluorescent resorufin mainly by mitochondrial and cytosolic enzymes, while dead cells rapidly lose this capacity once their membrane integrity has been compromised [70]. All tested formulations resulted in favorable toxicity profiles, even at the higher N/P ratio of 15, as no considerable influence on cell viability was observed after PX treatment (Fig. 5).

A very low but still negligible effect on cell viability was demonstrated solely by b-PEI PXs at N/P 15 (97.82%) and ApoE NM_0.2_/CP_0.8_ PXs at N/P 5 (99.28%). The positive control of 25% DMSO resulted in a survival rate of 55.54%. Summing up, these data showed that neither b-PEI PXs nor NM_0.2_/CP_0.8_ PXs with or without ApoE coating are expected to have a noticeable effect on U87 cell viability and, therefore, are well tolerated in in vitro experiments. As nylon-3 polymers have been investigated in order to design an advanced delivery system with excellent compatibility, our results are in line with previous studies that revealed high tolerability combined with efficient transfection efficacies especially for highly hydrophobic polymers [42, 64]. The functionalization of the NM_0.2_/CP_0.8_ PXs with ApoE was shown to not affect the cell tolerability in a significant manner. Although broadly used high molecular weight b-PEI is known for its high cellular toxicity due to its high cationic charge density [68], our findings demonstrated that b-PEI PXs do not exhibit significant cellular toxicity in our specific experimental setting. Altogether, these observations are especially important for future in vivo experiments as they demonstrate that our delivery systems are well tolerated after internalization into cells.

##### Hemocompability of polymers evaluated by hemolytic and erythrocyte aggregation assay

**Fig. 6:**
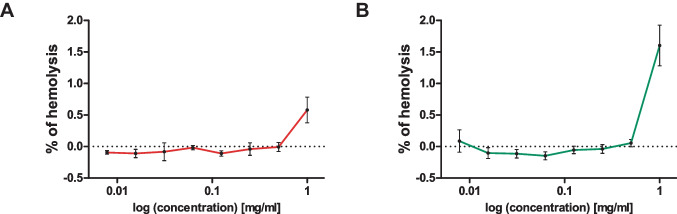
Hemolysis of human erythrocytes at pH 7.4 induced by b-PEI (**A**) and NM_0.2_/CP_0.8_ polymers (**B**) as a function of log concentration values (results are shown as mean ± SD as percentage of hemolysis in comparison to Triton-X treated cells representing 100% hemolysis, Hb0 represents the amount of basal hemoglobin found in the negative control samples, *n* = 3)

The hemocompability of b-PEI and NM_0.2_/CP_0.8_ polymers was investigated by hemolysis and erythrocyte aggregation assays. These assays are an indispensable initial step in evaluating the blood compatibility of NPs in advance of administering the materials intravenously in animals or humans. Several studies have reported a good correlation between the results of in vitro hemolysis assays and in vivo toxicity studies identifying hemolysis as a toxic effect [71]. Hemolysis occurs due to disruption of erythrocytes leading to the release of intracellular components such as hemoglobin, which can be measured spectroscopically. We tested 1 mg/ml as the highest polymer concentration assuming that the test concentration in vivo will not exceed this concentration. For comparison, in in vitro studies polymer concentrations of 0.011 mg/ml of b-PEI polymer to obtain PXs with N/P 7 and 0.203 mg/ml of NM_0.2_/CP_0.8_ polymers to obtain PXs with N/P 5 were used. The results of the hemolysis assays are represented in Fig. 6 and show that both formulations revealed no hemolysis at concentrations from 0.5 to 0.00781 mg/ml as the measured values were in the same range as the Hb0 value that represents the amount of basal hemoglobin found in the negative control sample. As shown in Fig. 6A, b-PEI polymer displayed slight hemolytic activity at the highest concentration of 1 mg/ml, namely a final hemolysis of 0.58%. At the same polymer concentration, as shown in Fig. 6B, NM_0.2_/CP_0.8_ polymers exhibited slightly higher hemolysis of 1.60%. Nevertheless, these values are still tolerable since substances are classified as non-hemolytic when hemolysis remains below 2% [72].

**Fig. 7:**
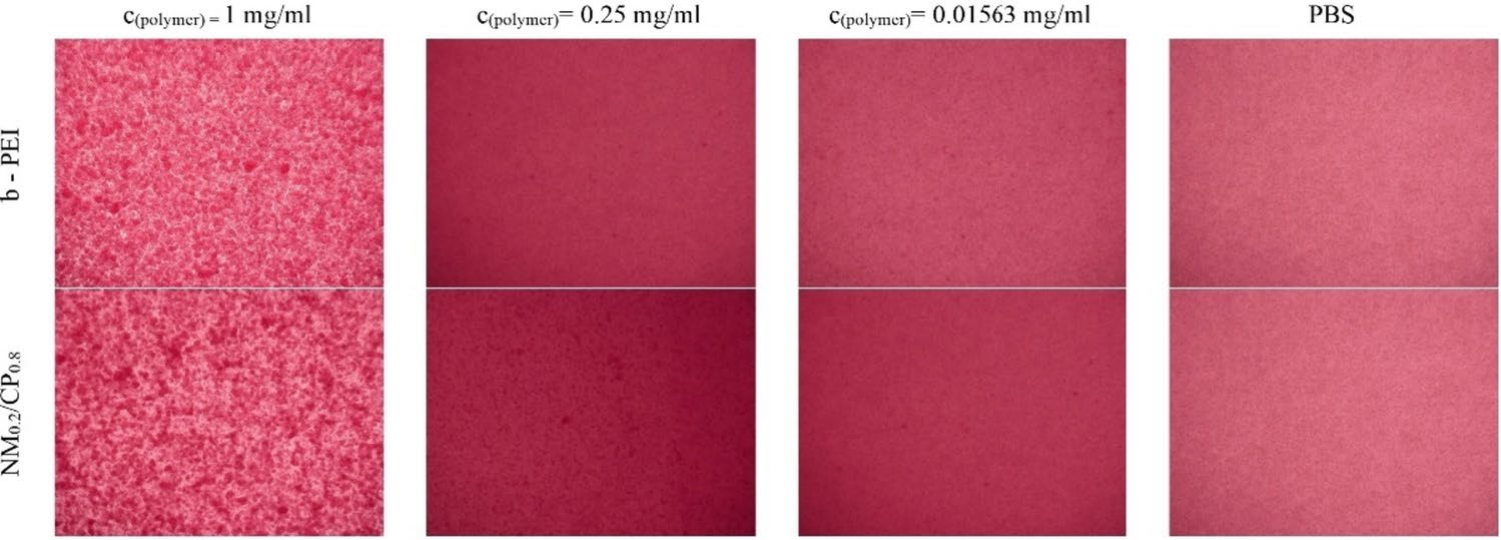
Erythrocyte aggregation profiles exemplarily shown for b-PEI and NM_0.2_/CP_0.8_ polymer concentrations of 1 mg/ml, 0.25 mg/ml and 0.01563 mg/ml in comparison to PBS used as negative control

The erythrocyte aggregation assay allowed only semiquantitative estimations about the hemocompatibility of the polymers. Results in this study are displayed in Fig. 7. Microscopic pictures of the erythrocytes are exemplarily shown for the concentrations of 1 mg/ml, 0.25 mg/ml, and 0.01563 mg/ml for b-PEI and NM_0.2_/CP_0.8_ polymers, as well as for PBS as negative control. PBS control samples did not induce any aggregation of the erythrocytes, whereas polymer solutions, depending on the concentration, caused a slight (0.25 mg/ml and 0.01563 mg/ml) to strong (1 mg/ml) aggregation of the RBCs, respectively.

These results suggest that a higher amount polymer and thus positive charges induced stronger aggregation effects due to interactions with negatively charged components of the RBC membrane. Additional hydrophobic interactions with the CP subunits of the NM_0.2_/CP_0.8_ polymers might lead to slightly more aggregation effects as visible for NM_0.2_/CP_0.8_ polymer concentrations of 0.25 mg/ml and 1 mg/ml. Taken together, the favorable hemolytic profiles and the low tendency to induce erythrocyte aggregation indicated that both polymers are well tolerated by RBCs, emphasizing the safe profile of these materials with regard to following in vivo experiments.

### In vivo biodistribution experiments

**Fig. 8:**
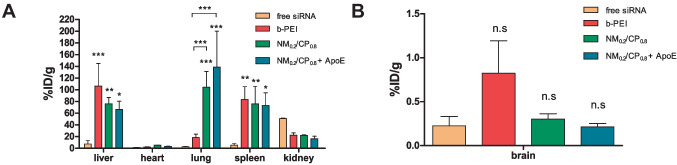
Biodistribution of ^177^Lu-labeled siRNA, b-PEI PXs, NM_0.2_/CP_0.8_ PXs and ApoE-NM_0.2_/CP_0.8_ PXs in dissected organs (**A**) and the brain (**B**) of SWISS mice 1 h post injection (results are presented as % ID/g and shown as mean ± SD, *n* = 3, two-way ANOVA with Bonferroni post hoc test, n.s not significant; *p* > 0.05, ^*^p < 0.05, ^**^p < 0.01, and.^***^p < 0.005, mice that urinated during the incubation period were excluded from the study to avoid falsification of the results due to undefined loss of radioactive material)

After it was shown that ApoE-NM_0.2_/CP_0.8_ PXs in particular mediated targeted and efficient cellular uptake and in vitro knockdown in combination with favorable toxicity profiles, they were subsequently investigated in vivo in SWISS mice to evaluate their biodistribution behavior compared to non ApoE-coated NM_0.2_/CP_0.8_ PXs and in addition to b-PEI PXs. Therefore, siRNA was covalently coupled with DTPA to enable the labeling with ^177^Lu as radioactive marker for biodistribution studies of formulations following an adjusted protocol previously described for ^111^Indium-labeling of siRNA [49]. ApoE-NM_0.2_/CP_0.8_ PXs, NM_0.2_/CP_0.8_ PXs, and b-PEI PXs were formed with ^177^Lu-radiolabeled siRNA and intravenously administered through the tail vein, and biodistribution was investigated 1 h post injection in comparison to ^177^Lu-labeled free siRNA as control. As measured by gamma scintillation counting of resected organs (Fig. 8A), free siRNA exhibited a different biodistribution profile in comparison to PXs and accumulated preferentially in the kidney (50.65% ID/g), as reported earlier [50]. Small amounts of siRNA were in addition found in the liver (7.44% ID/g) and in the spleen (5.48% ID/g ratio). The results indicated that ^177^Lu-labeled siRNA encapsulated with b-PEI accumulated mainly in the liver (106.41% ID/g) and spleen (83.08% ID/g), which is in good agreement with published data [73]. In contrast, with ^177^Lu-labeled siRNA encapsulated with NM_0.2_/CP_0.8_ was preferentially detected in the lung, as 104.41% ID/g and 138.87% ID/g were found for NM_0.2_/CP_0.8_ and ApoE-NM_0.2_/CP_0.8_ PXs, respectively. In addition, NM_0.2_/CP_0.8_ and ApoE-NM_0.2_/CP_0.8_ complexed siRNA accumulated, although to a smaller extent, in the spleen (NM_0.2_/CP_0.8_ PXs 75.74% ID/g and ApoE-NM_0.2_/CP_0.8_ PXs 72.98% ID/g) and in the liver (NM_0.2_/CP_0.8_ PX 75.92% ID/g and ApoE-NM_0.2_/CP_0.8_ PXs 66.54% ID/g ratio). As illustrated in Fig. 8B, only low concentrations of free ^177^Lu-labeled siRNA and complexed with both, b-PEI and NM_0.2_/CP_0.8_ were detected in the brain. In fact, the lowest radioactive signal in the brain was observed in mice treated with ApoE-coated NM_0.2_/CP_0.8_ PXs 1 h post injection.

Several in vivo studies performed with polyplexes have shown that a variety of parameters influence the biodistribution of siRNA formulations. Physicochemical properties of the NPs such as particle size, surface charge, and surface hydrophobicity further determine the stability of the polymer-siRNA complexes as well as interactions with proteins within the blood stream. Protein corona formation in turn can lead to recognition of the particles by the reticuloendothelial system (RES) determining the fate of the particles in the body after intravenous injection. Free labeled siRNA administered to mice was shown to be distributed to kidneys and the liver within minutes after injection, and a minor fraction of the siRNA was rapidly excreted via the urine so that levels of siRNA within the body decreased markedly after 24 h [74]. It is also known that siRNA is rapidly degraded by serum nucleases upon injection [75] implying that small fragments bearing the label might additionally circulate in the blood pool and show a different behavior than macromolecules, which needs to be considered as well when interpreting biodistribution data. Nevertheless, it was previously ascertained that renal clearance might occur more rapid than the degradation processes [76]. Moreover, free siRNA did not selectively accumulate in the other organs or in the brain, reflecting the poor ability of siRNA to penetrate the blood–brain barrier [50]. Taken together, our data for the biodistribution of ^177^Lu-labeled free siRNA in mice were consistent with previous reports. Labeled siRNA complexed with PEI demonstrated accumulation in the liver and the spleen. This goes in line with earlier findings, which suggest that PEI-particles are cleared from the blood stream after opsonization very rapid by phagocytosing macrophages ending up in the organs of the RES, namely, the liver and spleen [77]. In a double labeling approach in which both the siRNA and the polymer PEI were labeled with a radioactive marker via DTPA coupling to investigate the in vivo stability of the polyplexes, it was found that the polyplexes were taken up into the liver as a whole, but dissociation subsequently occurred during liver passage, resulting in the accumulation of free siRNA in the interstitium while the polymer was taken up into hepatocytes [73, 78]. Our data underpins these assumptions, as b-PEI-encapsulated ^177^Lu-labeled siRNA was detected mainly in the liver and the spleen. Small amounts of ^177^Lu-labeled siRNA detected in the kidney after b-PEI PXs injection can possibly be explained by disintegration of the b-PEI-siRNA complex within the blood stream leading to free siRNA, which consequently undergoes renal clearance. A somewhat different picture emerged in the case of ^177^Lu-labeled siRNA encapsulated with NM_0.2_/CP_0.8_ polymers, as it was detected in the liver and spleen to a smaller extent than b-PEI PXs siRNA, but to a higher extent in the lungs. Therefore, we suggest that a lower proportion of the NM_0.2_/CP_0.8_ PXs was recognized after opsonization by RES cells leading to internalization in the liver and spleen. A deposition in the lung was so far associated with the formation of aggregates due to interactions of polyplexes with blood components that subsequently get entrapped in the capillary bed of the lung [79]. In addition, it was shown that modification of particles with hydrophilic substances such es poly(ethylene glycol) (PEG) led to significantly decreased interactions with RBCs [73]. More hydrophobic NPs, as it is the case for NM_0.2_/CP_0.8_ PXs, seem to interact more easily with erythrocytes, what is also supported by the data of the erythrocyte aggregation assay described above, leading to accumulation of RBC-PX aggregates in the lung capillaries. Even though this effect was incisive, it did not lead to lethality of mice due to embolism incidents as described in previous investigations of biodistribution and kinetics of PXs [79, 80]. Against our expectations, no significant differences in biodistribution profiles, including no selective accumulation in the brain, were observed for NM_0.2_/CP_0.8_ PXs and ApoE-coated NM_0.2_/CP_0.8_ PXs. Subsequently, neither direct ApoE coating of NM_0.2_/CP_0.8_ PXs nor potential adsorption of ApoE from the blood to hydrophobic subunits of the NM_0.2_/CP_0.8_ PXs achieved selective or efficient siRNA delivery to the brain in this specific experimental setting at the time point investigated. One important aspect is related to potentially different pharmacokinetics of the various formulations injected. From our data, we conclude that while ApoE was shown to bind in particular to NM_0.2_/CP_0.8_ PXs, leading to successful internalization in LDL/LRP-positive glioblastoma cells, used as model cell line in vitro, it did not lead to brain accumulation of the PXs in vivo within 1 h*.* Therefore, we assumed that interactions between ApoE and PXs might not be stable enough in the biological environment of an in vivo setup and that the targeting ligand may be shed in the blood stream after injection before the system can reach the brain. PXs made of b-PEI, on the other hand, resulted in a seemingly but not statistically higher deposition within the brain. This deposition must be understood as a non-specific and untargeted accumulation of highly charged polyplexes, which do not reach the brain efficiently but deposit approximately 1% ID/g in the brain due to strong interactions with membranes. To address this issue, we propose to develop a covalent coupling protocol of polymers with ApoE, as it was for example already described for HAS NPs in previous studies [41, 81]. It is likewise conceivable, that the ApoE concentration chosen for direct coating of PXs, following previous publications with solid NPs [20], was too low to achieve efficient brain targeting effects in vivo with b-PEI and NM_0.2_/CP_0.8_ PXs and needs to be further optimized in future experiments. Taken together, the results pointed out that overcoming the blood–brain barrier in vivo remains a major bottleneck for siRNA delivery into the brain and that precisely designed delivery systems are needed for active targeting that fulfill the complex requirements present under physiological conditions. Specifically, in addition to appropriate properties of the delivery system, also sufficient residence in the circulation is required to allow interactions with respective receptors in target sites and thus uptake into targeted tissues.

## Conclusion

In conclusion, ApoE-NM_0.2_/CP_0.8_ PXs in particular yielded promising in vitro data regarding the possibility of selective brain targeting. However, biodistribution studies performed with PXs containing ^177^Lu-labeled DTPA-siRNA in mice were not consistent with in vitro results as virtually no radiolabeled siRNA was detected in the brain for any tested formulation. Overall, this study laid the foundation for investigations regarding selective siRNA delivery with PXs into the brain using the ApoE approach. PS80 precoating is not appropriate for ApoE adsorption on PXs, but this limitation can potentially be tackled by modifying polymers with hydrophobic modifications for enhanced ApoE binding. Nevertheless, since the in vitro*-*in vivo correlation was very poor, we suggest that, among other factors, the loose attachment of ApoE is not durable within complex biological environments and that more stable covalent linkage approaches for PXs should be addressed in future experiments. Furthermore, this study provides evidence for the need of appropriate in vitro models that better simulate physiological conditions and therefore provide more accurate estimates regarding the behavior of delivery systems in vivo. Ongoing work currently focuses on the establishment of a human BBB co-culture model mimicking the neurovascular unit using induced puri- and multipotent stem cells to provide a sophisticated tool for the development of effective drug delivery systems for CNS delivery in the future.

### Supplementary Information

Below is the link to the electronic supplementary material.

Supplementary file1 (DOCX 140 KB)

## Data Availability

The datasets generated and analyzed during the current study are available from the corresponding author on reasonable request.
