# Supplementary material for: ApoE—functionalization of nanoparticles for targeted brain delivery—a feasible method for polyplexes?
Source: Drug Deliv Transl Res. 2023 Dec 12;14(6):1660–77. doi: 10.1007/s13346-023-01482-w (PMC11052808; doi:10.1007/s13346-023-01482-w)
Supplement: Supplementary file 1 — Supplementary file1 (DOCX 140 KB) [file 13346_2023_1482_MOESM1_ESM.docx]

**Supplementary material**

1. **Size and Zeta Potential Analysis of PXs by Dynamic Light Scattering and Laser Doppler Anemometry**

1.1 PS80-concentration optimization

| **A** |  | **B** |  |
| --- | --- | --- | --- |

**Figure S1.** DLS measurements of PS80-b-PEI PXs (**A**) and PS80-NM_0.2_/CP_0.8_ PXs (**B**) coated with varying PS80 concentrations (0.01% - 0.5% PS80) in comparison to uncoated particles (0% PS80). (Data points indicate mean ± SD, n = 3).

1. **Quantification of cellular uptake into glioblastoma cells by flow cytometry**

2.1 LRP1-Receptor expression of glioblastoma cells

**Figure S2.** LRP1-receptor expression of U87 glioblastoma cells as determined by flow cytometry and presented as median fluorescence intensity (MFI): Cells were first stained with anti – LRP1 primary antibody or mouse IgG1 monoclonal antibody as isotype control (IC) to exclude unspecific binding and consequently reacted with AF488-labeled secondary goat anti-mouse IgG H&L antibody. Blank samples were left unstained. (Results are presented as median fluorescence intensity (MFI) and shown as mean ± SD, n = 3, one-way ANOVA with Bonferroni post-hoc test, *** p < 0.005).

2.2 Trypan quenching

**Figure S3.** Cellular uptake of uncoated PXs and ApoE coated PXs after 24 h incubation as quantified by flow cytometry performed with and without trypan quenching and presented as median fluorescence intensitiy (MFI). Negative control: untreated cells (blank) and with free siRNA treated cells. (Results are shown as mean ± SD, n = 3, two-way ANOVA with Bonferroni post-hoc test, n.s (= not significant) p > 0.05, * p < 0.05, *** p < 0.005).

3. Influence of PS80 coating on celllar uptake efficacy

**Figure S4.** Cellular uptake of uncoated b-PEI and PS80-b-PEI PXs (b-PEI PXs: N/P ratio 7, final PS80 concentration = 0.1%) after 24 h incubation as quantified by flow cytometry and presented as median fluorescence intensitiy (MFI). Negative control: untreated cells (blank) and with free siRNA treated cells. Positive control: Lipofectamin 2000 lipoplexes (LF). (Results are shown as mean ± SD, n = 3, one-way ANOVA with Bonferroni post-hoc test, n.s (= not significant) p > 0.05, *** p < 0.005).
